# Effectiveness of a Food Supplement Based on Glucomannan, D-Chiro-Inositol, *Cinnamomum zeylanicum* Blume and Inulin in Patients with Metabolic Syndrome

**DOI:** 10.3390/nu16020249

**Published:** 2024-01-12

**Authors:** Roberto Citarrella, Roberta Chianetta, Simona Amodeo, Luigi Mirarchi, Anna Licata, Maurizio Soresi, Nicola Veronese, Mario Barbagallo, Lydia Giannitrapani

**Affiliations:** 1 Department of Health Promotion Sciences, Maternal and Infant Care, Internal Medicine and Medical Specialties (PROMISE), University of Palermo, 90127 Palermo, Italy; roberto.citarrella@unipa.it (R.C.); roberta.chianetta@you.unipa.it (R.C.); simona.amodeo@unipa.it (S.A.); anna.licata@unipa.it (A.L.); maurizio.soresi@unipa.it (M.S.); nicola.veronese@unipa.it (N.V.); 2 Department of Internal Medicine, National Relevance and High Specialization Hospital Trust ARNAS Civico, Di Cristina, Benfratelli, 90127 Palermo, Italy; luigi.mirarchi@arnascivico.it; 3 Institute for Biomedical Research and Innovation (IRIB), National Research Council, 90146 Palermo, Italy

**Keywords:** metabolic syndrome, T2DM, glucomannan, D-chiro-inositol, *Cinnamomum zeylanicum* blume, inulin

## Abstract

Metabolic syndrome (MetS) is associated with cardiovascular risk factors, such as insulin resistance, dyslipidaemia, hypertension and abdominal obesity. Given the growing need to investigate food supplements with positive health effects, this study was aimed at testing the benefits of a specific supplement for people with MetS. Fifty-eight subjects with MetS and T2DM or impaired glucose tolerance assuming metformin, were randomly assigned to take a food supplement of glucomannan, D-chiro-inositol, *Cinnamomum zeylanicum* blume and inulin at a daily fixed dose of 4 g orally for four months. Body weight, waist circumference, plasma lipid profile (total cholesterol, LDL, HDL and triglyc-erides), plasma glycaemic profile and visceral adiposity index (VAI) were measured at baseline and after four months of supplementation. After 16 weeks, in subjects with T2DM or insulin resistance who took the supplement (+ metformin), there was a significant reduction in body weight and BMI (*p* < 0.0001), serum insulin (*p* < 0.05) and the HOMA index (*p* < 0.01), as well as in the lipaemic pattern, with a significant improvement in total serum cholesterol (*p* < 0.005), triglycerides (*p* < 0.03) and LDL (*p* < 0.02). Our study shows that the food supplement tested is a valid and safe alternative therapeutic approach in the management of MetS and all its resulting risk factors, as its efficacy has been demonstrated across anthropometric, glucose, lipid and hepatic parameters.

## 1. Introduction

Metabolic syndrome (MetS) is a condition characterized by the simultaneous presence of haemodynamic, anthropometric and metabolic variations that predispose a person to a high risk of developing cardiovascular diseases and type 2 diabetes mellitus (T2DM). Its origin is attributable to a high-calorie diet, rich in simple sugars and saturated fats, coupled with a sedentary lifestyle in genetically predisposed individuals [1].

Obesity, the principal condition related to MetS, has increased its prevalence worldwide in the past 50 years, thus reaching pandemic levels. This is why it represents a major health challenge because it substantially increases the risk of chronic diseases such as T2DM [2].

The management of MetS, besides pharmacologically treating the individual components, includes cornerstones such as healthy eating (especially the Mediterranean Diet, MD), regular exercise and weight loss [3,4], in a multidisciplinary approach that seeks to modify the patient’s lifestyle; obviously, such an approach requires a team of medical and health specialists together with behavioral psychology and food education professionals (such as dieticians and nutritionists) [5].

In fact, the introduction from the late 50 s of ultra-processed foods (UPFs), have become a significant, and in some cases the main, source of dietary energy not only in high-income countries but also in low-and middle-income countries, involving a shift away from traditional diets towards those linked with obesity and diet-related non-communicable diseases (NCDs) on a truly global scale during the current era [6].

However, even after choosing the best available treatment for the underlying pathologies and undergoing counselling for lifestyle changes, poor compliance often leads to poor results. Consequently, it has become necessary to identify new compounds and/or more effective treatments in order to have higher efficacy and fewer side effects. An alternative approach could be the use of natural agents, which provide an excellent contribution to modern therapies. Today the growing demand for natural products to complement conventional therapies has led to the birth of a new scientific discipline called Nutraceuticals, from the fusion of the terms “nutrition” and “pharmaceutical” [7].

Besides the fact that the supplements cannot be the substituent of a normal, balanced diet, today’s nutraceutical approach presents a valid and safe choice for the management of patients with MetS, though in the case of particularly severe conditions, drug therapy cannot be replaced by nutraceutical treatment. Very often, however, nutraceuticals can be used in association with the therapies prescribed to better modulate the therapeutic effects of drugs, modulating their dosage and reducing the risk of complications. For example, turmeric, chlorogenic acid, cinnamon and fibres such as glucomannan and inulin have shown important benefits in patients with MetS in combination with drug therapies. In particular, glucomannan, inulin and cinnamon are among the substances studied that have an established inverse association with T2DM, hypertriglyceridaemia, hypercholesterolaemia and obesity [8].

Glucomannan, is a hydrocolloidal dietary fiber with several healthy properties which include lowering of cholesterol, triglycerides, glycemia, blood pressure and anti-diabetic and anti-obesity activity. Thanks to these effects it has been offered as a food additive as well as a dietary supplement in many countries and has been the subject of numerous clinical and experimental studies to investigate these properties [9].

D-chiro-inositol (DCI) is a form of inositol produced by many plants that have insulin-like properties, acting as secondary messengers in insulin signal transduction. Recent studies in diabetic mice model have demonstrated its involvement in cellular glucose uptake in skeletal muscle and insulin secretion in mouse islets cells. It is currently used alone or in association with drug therapies in the treatment of polycystic ovary syndrome (PCOS), and T2DM [10].

*Cinnamomum zeylanicum* blume plant, and its active ingredients have been shown to regulate glycemia with insulin-mimetic properties, anti-diabetic, anti-oxidant and anti-inflammatory effects, as well as beneficial effects on the lipid profile [11,12,13].

Inulin, a water-soluble dietary fibre which has been recently approved by the Food and Drug Administration for improving the nutritional values of food products, belongs to the class of prebiotic non-digestible food components and, being well tolerated by diabetics, it has been used in these patients to reduce serum lipids, control hunger and help reduce weight by modulating glucagon-like peptide [14].

The primary objective of our study was to evaluate whether 4 months of administration of a supplement based on glucomannan, D-chiro-inositol (DCI), *C. zeylanicum* blume and inulin as an add-on to the patient’s diet, could improve anthropometric and cardio-metabolic parameters (weight, BMI and waist circumference) in subjects with T2DM or reduced glucose tolerance, compared to a group following standard therapy.

## 2. Materials and Methods

### 2.1. Participants

Our ambulatory is dedicated to patients affected by different metabolic disorders, mainly type I and type II diabetes, metabolic syndrome, obesity. Approximately, about 500 patients each year are visited.

Fifty-eight patients (M/F), followed up as outpatients at the Metabolic Diseases and Cardiovascular Prevention Clinic, Internal Medicine Unit, P. Giaccone University Hospital in Palermo, Italy, were consecutively enrolled to this study. We included patients who were over 18 years old, able to understand and sign the informed consent, overweight, and who had a diagnosis of MetS according to the National Cholesterol Education Program Adult Treatment Panel III (ATP III) criteria [1]; we excluded patients having life-expectancy of less than 6 months (e.g., severe renal failure, hepatic failure or similar), HIV, HBV, HCV infection, any malignant tumor or who were pregnant or planning to become pregnant in the following 6 months. The study was approved by the Local Ethics Committee during the 15 January 2020 session (number 1/2020) as a part of a wider study on MetS.

The eligible subjects, after having read and signed the informed consent, underwent a medical examination, with a careful collection of family and personal medical history (concerning cardio-metabolic risk factors and any previous cardio- and cerebrovascular events), pharmacological and nutritional medical history (concerning lifestyle, in particular eating habits and physical activity).

The patients recruited into the study were evaluated twice: at time 0 (time of administration of the intervention) and after 4 months. At each assessment, in both groups, the following were detected: anthropometric parameters (weight and height, with subsequent calculation of BMI kg/m^2^); measurement of waist circumference (cm); blood chemistry parameters: glycemics (fasting blood sugar, insulinemia, HbA1c), lipids (total cholesterol, LDL-C, HDL-C, triglycerides), hepatic (AST, ALT), renal (creatininemia, with eGFR calculation), indices: VAI (Visceral Adiposity Index), HOMA-IR (Homeostatic Model Assessments of Insulin Resistance).

### 2.2. Study Design

This was an observational, prospective study with two arms: one treated with oral supplementation of DCI, glucomannan, *C. zeylanicum* blume and inulin (GludiaTM), and the other following a standard care plan. After the baseline visit, the treated group took two sachets of the oral supplement every day, 30 min before lunch and dinner; the supplement was composed of glucomannan 1000 mg, DCI 40 mg, *C. zeylanicum* blume 250 mg and inulin 200 mg in a formulation of 4 g total each day. The control group followed standard care for T2DM. No oral supplements for improving cardiometabolic profiles were permitted in either group during the follow-up time. During the 16 weeks of follow-up, the patients were contacted by phone every 30 days to evaluate their adherence to the treatment and possible side effects. After the 16-week follow-up period, the patients in the treated group were visited by the same physician as for the baseline examination.

### 2.3. Outcomes

Several outcomes of cardiometabolic health were examined:

- Anthropometric measures, such as weight, height and waist circumference were measured by a trained dietician. Visceral adiposity index (VAI) was calculated using a combination of waist circumference, body mass index (BMI), triglycerides and high-density lipoproteins (LDL), using the equations proposed in [15].
- Laboratory measures included markers of insulin-resistance or glucose metabolism, such as glycosylated haemoglobin (HbA1c), fasting plasma glucose and serum insulin. We also calculated the HOMA index [16] based on fasting plasma glucose and serum insulin levels at 8 am. HOMA uses fasting measurements of blood glucose and insulin concentrations to calculate indices of both insulin sensitivity and β-cell function. The principle of HOMA is that blood glucose and insulin concentrations are related by the feedback of glucose on β-cells to increase insulin secretion. Finally, we evaluated cholesterol metabolism, renal and hepatic function. All the laboratory measurements were performed using standard laboratory assessments.

### 2.4. Statistical Analysis

To calculate the sample size, we hypothesized that, by determining a type I error of 5% and a type II error of 20%, a sample size of 28 (14 for each group) would be sufficient to reveal a difference between the treated and control groups of 3 kg (with a standard deviation of 4).

Baseline characteristics were compared between the treatment and control groups using independent t tests, chi-square tests, or Fisher’s exact test, as appropriate, i.e., when the count in one cell for 2 × 2 table was less than 5. Matched *t* tests were used for within group comparisons of baseline and 16-week data, and changes were calculated as the difference between the two values (delta). Comparisons between data for the groups at 16 weeks were computed by using a generalized linear model, adjusted for the baseline value of the corresponding test. Data were also reported as percentages calculated as the difference between follow-up values vs. baseline values divided for baseline data in both groups, for graphical reasons. Significance was accepted if *p* < 0.05, and all tests were two-tailed. All analyses were performed using SPSS 25.0 for Windows (IBM SPSS) and the graphs were executed using Microsoft Excel.

## 3. Results

Overall, 58 patients affected by MetS and T2DM or impaired glucose tolerance were enrolled. The main reasons of exclusion were the absence of the conditions needed for the study and the lack of interest for the study or the presence of renal failure. Their mean age was 55.9 ± 16.5 years (range: 20–83), and they were prevalently female (60.3%).

Table 1 shows the baseline characteristics of the patients, divided into treatment (n = 29) and control (n = 29) groups. The group treated with the oral supplement of DCI, glucomannan, *C. zeylanicum* blume and inulin was significantly younger than the control group (*p* = 0.006), whilst no significant difference was detected for the percentage of females (*p* = 0.29). Patients in the treated group reported significantly lower VAI (*p* = 0.02) (Table 1) at the baseline evaluation compared to the control group. The patients treated with the oral supplement reported lower triglyceride levels (*p* = 0.002) than controls at baseline. No other significant differences emerged between the two groups at baseline.

**Figure 1:**
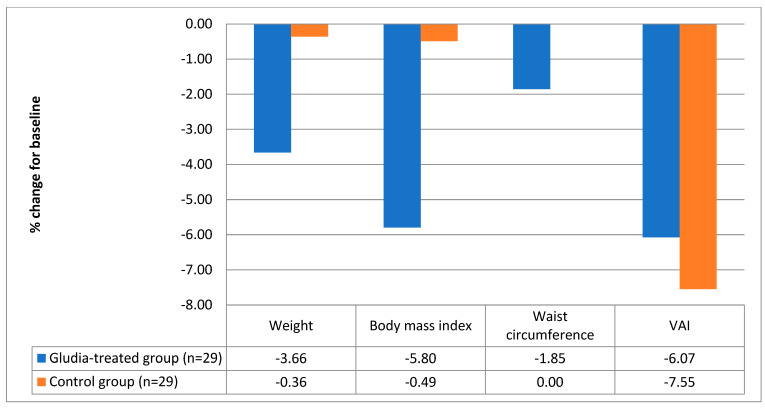
Variations in the main anthropometric parameters in the treated and in the control group [weight (−3.66 vs. −0.36 kg), body mass index (−5.80 vs. −0.49 kg/m^2^), waist circumference (−1.85 vs. 0 cm) and VAI (−6.07 vs. −7.55)].

**Figure 2:**
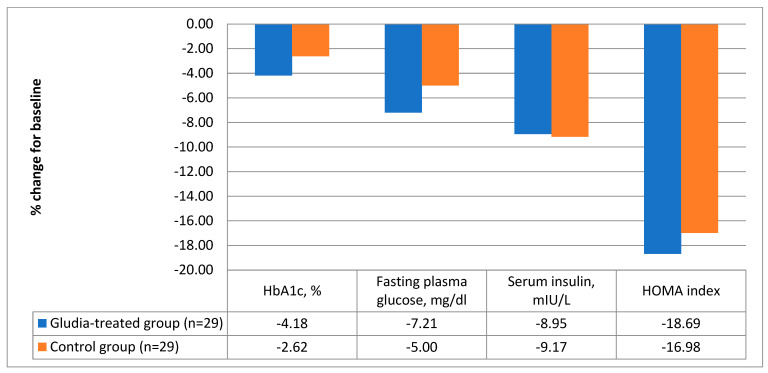
Variations in the main glucometabolic parameters in the treated and in the control group [(HbA1c (−4.18 vs. −2.62%), fasting plasma glucose (−7.21 vs. −5.00 mg/dL), serum insulin (−8.95 vs. −9.17 mIU/L) and HOMA index (−18.69 vs. −16.98)].

**Figure 3:**
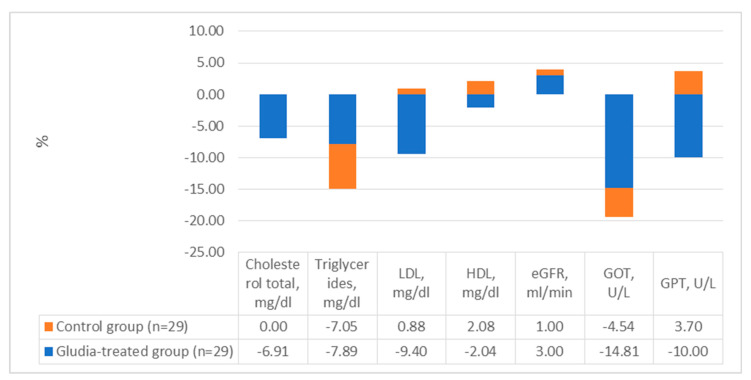
Variations in lipid profile, kidney and liver function tests in the case study.

After 16 weeks of treatment, as shown in Table 1, patients treated with the oral supplement reported significantly better values in body weight (delta = −3.4 ± 3.9 kg; a decrease of 3.66% compared to the baseline value, *p* < 0.0001, Figure 1) and BMI (delta = −2.0 ± 3.4 kg/m^2^; *p* < 0.0001). Furthermore, patients treated with the supplement reported a significant decrease in serum insulin (delta = −1.7 ± 4.4 mIU/L; *p* = 0.049) and in the HOMA index (delta = −1.00 ± 2.00; *p* = 0.01). The data are graphically reported in Figure 2. Finally, treatment with DCI, glucomannan, *C. zeylanicum* blume and inulin was associated with a significant improvement in total serum cholesterol (delta = −13 ± 22 mg/dl; variation in %: −6.91 compared to baseline values; *p* = 0.005), triglycerides (del-ta = −9 ± 21 mg/dl; variation in %: −7.89 in the treated group compared to baseline; *p* = 0.03) and LDL (delta = −11 ± 23 mg/dl; variation in %: −9.40 in the treated group vs. baseline; *p* = 0.02) (Table 1 and Figure 3).

Furthermore, we compared the mean differences between the treated and control groups after adjusting the follow-up data for the corresponding baseline values and mean age. The patients treated with the oral supplement had a significant decrease compared to the controls in body weight (mean difference = −2.7; 95%CI: −4.4 to −1.1; *p* = 0.001) and BMI (−0.98 kg/m^2^; 95%CI: −1.59 to −0.37; *p* = 0.002) (Table 1). Finally, treatment with the supplement was associated with a better profile of cholesterol parameters since, compared to controls, it was associated with a significant reduction in serum cholesterol levels (mean difference = −13 mg/dL; 95%CI: −25 to −1; *p* = 0.03) (Table 1).

In the treated group, two patients were lost during the follow-up: one without giving a reason; the other for gastrointestinal issues (stomach pain) that may have been attributable to the supplement.

## 4. Discussion

The wide spreading of obesity world-wide, has become a major health challenge because of its link to cardiovascular risk factor such as T2DM. From a medical healthcare point of view as a consequence, the MetS and T2DM pandemic reduction is the highest priority intervention [2].

Recently, the nutraceutical approach has been suggested as a safe and efficient strategy to support therapeutic efforts in the management of patients with MetS [7]. There are several classes of nutraceuticals that have been used to obtain benefits in treating MetS, hypertension and T2DM, thanks to their positive effects on weight reduction, post-prandial blood sugar, insulin resistance and inflammation [17].

The results of research on natural compounds have been warmly welcomed by the international scientific community and have contributed to the spread of the dietary model of the MD, to the point that in 2010 UNESCO included this diet in the prestigious list of Intangible Cultural Heritage of Humanity [18]. The MD is rich in particularly beneficial substances, such as polyphenols, phytosterols, ω-3 and MUFA/PUFA, fibre and vitamins [19]. This in contrast with the extensive use of UPFs whose greater contribution to total energy intake results in poorer dietary quality, higher risks of all-cause mortality, obesity, cardio-metabolic diseases, etc. [6].

Moreover, nutraceuticals have been used to prevent, support and treat some pathological conditions, because they can be considered as food-drugs, given that they possess the healing properties of natural active ingredients of recognized efficacy, are safe and can have better tolerability compared to drugs which can sometimes cause the onset of side effects. So, they can be effectively used by including them in the daily diet, since they are positioned in the space that goes “beyond the diet, before the drugs”.

Among those most studied, glucomannan, DCI, cinnamon and inulin seem the most ef-ficacious in treating T2DM, hypertriglyceridaemia, hypercholesterolaemia and obesity [9,20,21,22,23,24,25].

Glucomannan, a high molecular weight hydrocolloidal polysaccharide consisting mainly of glucose and mannose used as dietary fibre, in addition to regulating intestinal activity can reduce levels of glucose, cholesterol, triglycerides and blood pressure, promote weight loss and strengthen the immune system thanks to its adhesive properties [9,20]. In particular its ability to delay stomach emptying and adjust the rate of dietary sugar absorption by the small bowel, can be related to an insulin sensitivity action [26]. Moreover, recent studies have shown increase in serum high molecular weight adiponectin levels and decrease of plasma ghrelin, leptin and glucagon-like peptide-1 levels thus suggesting a role in improving insulin resistance [27]

DCI, one of the nine stereoisomeric forms of inositols, together with Myo-Ins is incorporated in the inositol phosphoglycan (IPG) of the cell membrane which is involved in insulin signal transduction. It is implicated in the storage of sugars as glycogen, in the regulation of the respiratory chain for the production of ATP, and in the control of insulin secretion by pancreatic β-cells [28].

Numerous clinical studies have shown that it has significant beneficial effects in PCOS, a syndrome that shares some common characteristics with MetS (e.g., insulin-resistance), where it shows an etiological role [21]. Recent studies suggest that inositol could be a valid strategy to improve glycaemic control in T2DM; in fact, its integration is effective in lowering both fasting glycaemia and HbA1c levels [22,23].

It also known that in DM2, also pancreatic α-cells present insulin resistance. Experimental studies, conducted on in vitro models (pancreatic α-TC1 clone 6 cells), have shown that DCI treatment prevents the insulin resistance of TC1-6 cells chronically exposed to palmitate restoring insulin-mediated glucagon suppression [29].

Another study on a mice model have investigated the effects and underlying molecular mechanisms of DCI on hepatic gluconeogenesis in mice fed a high-fat diet and saturated palmitic acid-treated hepatocytes. The authors described a novel pathway by which DCI prevents hepatic gluconeogenesis, reduces lipid deposition and ameliorates insulin resistance [30].

Cinnamon, obtained from the stem and roots of the *C. zeylanicum* blume (or verum) plant, and its active ingredients such as cinnamaldehyde, cinnamate, cinnamic acid and eugenol, in the form of aqueous and alcohol extracts, have a variety of therapeutic effects. In fact, the presence of biologically active substances regulates blood sugar with insulin-mimetic properties, which increase the absorption of glucose by triggering the activity of the insulin receptor kinase, the self-phosphorylation of the insulin receptor and glycogen synthetase activity with anti-diabetic, anti-oxidant and anti-inflammatory effects, as well as benefits for the lipid profile [11,12,13].

Inulin, instead, is an oligosaccharide present in various foods of plant origin (generally extracted from chicory and artichokes) and belongs to the class of prebiotic non-digestible food components that stimulate the proliferation of numerous beneficial bacteria in the colon [24]. It usually promotes digestion, reduces intestinal gas, improves the quality of the microbiota by increasing the percentage of eubiotic bacteria of the genus Bifidobacterium and decreasing the population of harmful bacteria [24], increases faecal mass and the number of evacuations, increases the absorption of calcium, iron and magnesium, is well tolerated by diabetics, reduces serum cholesterol and triglycerides, controls hunger and helps reduce weight by modulating glucagon-like peptide [14].

However, data from intervention studies in humans on the effectiveness of the main nutraceuticals in regulating glycemia, both in patients with T2DM and in individuals at risk of diabetes, are still few, sometimes conflicting, and, in general, carried out in small groups and for fairly limited periods of time and even if, in particular for some of them, the results seem suggestive of a possible anti-hyperglycemic effect, they must be confirmed by studies on larger and longer-term series before we can give precise recommendations for their usage.

In our study, after 16 weeks of treatment, subjects with T2DM or insulin resistance who took the supplement in addition to standard metformin therapy reported significantly better values in body weight and BMI. They also reported a significant decrease in parameters related to glycemic control like serum insulin and the HOMA index, and they experienced a significant improvement in total serum cholesterol, triglycerides and LDL values. These results, in particular the decrease in body weight and the significant reduction in serum cholesterol levels, were still significant when adjusted the follow-up data for the corresponding baseline values and mean age.

At the end of this open-label, two-arm prospective study, after 16 weeks of taking the natural supplement, positive effects on metabolic parameters, such as cholesterol profile, were recorded in accordance with previous clinical studies. Its administration has therefore shown numerous and different effects on the entire cardiometabolic picture, thus reducing these risk factors. It is already known that food supplemets may play a valuable role in the treatment of MetS, and we believe that with this study we have proved the importance that dietary changes can have by influencing the potential to develop T2DM as well as cardiovascular disease.

First of all, an improvement in anthropometric parameters was highlighted, with a significant decrease in body weight and BMI, consistent with the results obtained from studies in the literature. The present study also highlighted a significant reduction in fasting blood sugar and HbA1c, affirming the positive role of the supplement and its components on glycometabolic parameters. In particular, this effect on glucose metabolism had already been observed in studies conducted on women with T2DM in which daily oral administration of inulin had shown significant anti-glycaemic effects [14,24].

In vivo mice studies have also shown that glucomannan and inulin extracts, when combined, are more effective in decreasing blood glucose levels than the action of the single components [25]. Furthermore, the insulin-sensitizing and hypo-glycaemic effect of the supplement can be attributed to the presence of DCI, as in studies conducted on diabetic mice this component improved glucose metabolism and increased glucose uptake at skeletal muscle level, where it induced the translocation of GLUT4 [22]. In addition, DCI has been shown to induce insulin secretion in β cells and potentiate glucose-stimulated insulin secretion [23].

Finally, Cinnamon, as reported by Sartorius et al., showed the ability to reduce insulin resistance, lower blood glucose and serum lipid levels, and improve obesity-related T2DM in mice by activating both PPARγ and PPARα pathways [11,12,13].

Our study is consistent with the results of previous works, highlighting the possible role of a nutraceutical approach in the management of MetS and stressing the importance of using this approach in association with standard therapy to improve its efficacy with the lowest dose, and achieve the goal of more effectively controlling cardiovascular risk factors and their impact in terms of organ damage.

The findings of our study must be interpreted within certain limitations. Firstly, it was not a randomized controlled trial: therefore, we cannot exclude a potential selection bias. Secondly, the sample size, even if sufficient to reach the primary outcome, was relatively small since it should be approximately 1000 to reach a clinical significance. Thirdly, the follow-up lasted only 16 weeks, so other studies with a longer follow-up are needed.

## 5. Conclusions

Starting from the premises that supplements cannot be the substituent of a balanced diet and that their usefulness must always be accompanied by a change in lifestyle, especially with regards to physical activity, our study demonstrates the efficacy of our glucomannan, DCI, *C. zeylanicum* blume and inulin-based supplement on anthropometric, glucose and lipid parameters. In our experience, putting these components together could be an effective and safe therapeutic approach in the management of MetS. Our results are intended to contribute to the already existing scientific literature regarding the nutraceutical potential of glucomannan, DCI, *C. zeylanicum* blume and inulin, but in particular we want to give further support to patients, given the already proven role of the supplement as a valid alternative (in the early phases as a prevention), or as a complementary therapy in the treatment of MetS, a condition for which, currently, there are limited pharmacological approaches.

## Figures and Tables

**Table 1:** Participants’ mean baseline characteristics and outcomes at follow-up, by group.

	Gludia-Treated Group (*n* = 29)			Control Group (*n* = 29)			Between Groups (Change 16 Weeks vs. Baseline)	*p* Value between Groups ^a^
	Baseline	16 Weeks	Δ	Baseline	16 Weeks	Δ	Δ	
Measure	Mean ± SD	Mean ± SD	Mean ± SD	Mean ± SD	Mean ± SD	Mean ± SD	Mean (95%CI)	
Age, years	50 (19)	-	-	62 (11)	-	-	-	0.006
Females, %	51.7	-	-	69	-	-	-	0.29
Weight, kg	92.9 (24)	89.6 (24)	−3.4 ± 3.9 ^^^	82.9 (1.4)	82.6 (15.1)	−0.3 ± 2.0	−2.7 (−4.4 to −1.1)	0.001
Body mass index, kg/m^2^	34.5 (7.2)	32.4 (6.6)	−2.0 ± 3.4 ^^^	30.9 (5.6)	30.7 (5.9)	−0.15 ± 0.78	−0.98 (−1.59 to −0.37)	0.002
Waist circumference, cm	108 (18)	106 (16)	−2 ± 6	106 (16)	106 (15)	0 ± 3	−0.99 (−3.45 to 1.47)	0.42
VAI	4.28 (1.69) *	4.01 (1.50)	−0.26 ± 1.03	6.22 (3.95)	5.74 (3.84)	−0.47 ± 1.51	−0.10 (−0.79 to 0.59)	0.34
HbA1c, %	6.22 (1.06)	5.96 (0.73)	−0.26 ± 0.77	6.47 (0.59)	6.31 (0.58)	−0.17 ± 0.20 ^^^	−0.20 (−0.43 to 0.03)	0.09
Fasting plasma glucose, mg/dL	111 (32)	102 (23)	−8 ± 31	120 (21)	114 (19)	−6 ± 18	−8.19 (−18.23 to 1.85)	0.11
Serum insulin, mIU/L	19.0 (6.5)	17.4 (5.8)	−1.7 ± 4.4 ^	17.0 (5.7)	15.4 (5.4)	−1.6 ± 1.9 ^^	0.13 (−1.52 to 1.78)	0.82
HOMA index	5.35 (2.77)	4.35 (1.52)	−1.00 ± 2.00 ^^	5.24 (2.20)	4.37 (1.72)	−0.89 ± 1.21 ^^	−0.05 (−0.73 to 0.64)	0.88
Cholesterol total, mg/dL	188 (36)	175 (31)	−13 ± 22 ^^	192 (37)	192 (43)	0 ± 23	−13 (−25 to −1)	0.03
Triglycerides, mg/dL	114 (33) **	105 (30)	−9 ± 21 ^^	156 (62)	146 (61)	−11 ± 28	−4 (−19 to 9)	0.50
LDL, mg/dL	117 (35)	105 (30)	−11 ± 23 ^^	113 (34)	114 (39)	1 ± 25	−11 (−23 to 1)	0.08
HDL, mg/dL	49 (10)	48 (9)	−1 ± 5	48 (11)	49 (11)	1 ± 5	−1 (−4 to 2)	0.42
eGFR, ml/min	100 (22)	103 (21)	3 ± 11	102 (26)	101 (25)	−1 ± 10	−4 (−23 to 15)	0.69
GOT, U/L	27 (19)	23 (13)	−4 ± 9 ^	22 (9)	21 (9)	−1 ± 6	−1 (−5 to 2)	0.39
GPT, U/L	30 (19)	27 (18)	−3 ± 7 ^	27 (15)	28 (16)	1 ± 9	−2 (−8 to 4)	0.52

Notes: *: *p* < 0.05; **: *p* < 0.001 between Gludia and controls at the baseline evaluation compared using an independent Student’s *T* test; ^: *p* < 0.05; ^^: *p* < 0.001; ^^^: *p* < 0.0001 within changes (follow-up versus baseline) in the same group, compared using a matched Student’s *T* test; a *p*-values for between groups at the follow-up evaluations were calculated using a generalized linear model, adjusted for baseline values of the two groups.

## Data Availability

Data are available upon reasonable request to the corresponding author.
